# Assessment of degenerative states of articular cartilage via sparse laser-based mid-infrared attenuated total reflectance spectroscopy

**DOI:** 10.1016/j.ocarto.2026.100826

**Published:** 2026-05-30

**Authors:** P. Krebs, U. Blazhko, M. Nägele, V. Tafintseva, B. Zimmermann, V. Virtanen, E. Nippolainen, R. Shaikh, I.O. Afara, J. Töyräs, S. Saarakkala, A. Kohler, B. Mizaikoff

**Affiliations:** a Institute of Analytical and Bioanalytical Chemistry, Ulm University, Ulm, Germany; b Faculty of Science and Technology, Norwegian University of Life Sciences, Ås, Norway; c OptoPrecision GmbH, Bremen, Germany; d Research Unit of Health Sciences and Technology, Faculty of Medicine, University of Oulu, Oulu, Finland; e Department of Technical Physics, University of Eastern Finland, Kuopio, Finland; f Science Service Center, Kuopio University Hospital, Kuopio, Finland; g School of Electrical Engineering and Computer Science, The University of Queensland, Brisbane, Australia; h Department of Diagnostic Radiology, Oulu University Hospital, Oulu Finland; i Hahn-Schickard, Ulm, Germany

**Keywords:** Articular cartilage, Osteoarthritis, Arthroscopy, Infrared spectroscopy, Quantum cascade laser

## Abstract

**Objective:**

To investigate capability of sparse mid-infrared spectral data obtained by a prototype infrared attenuated total reflection (IR-ATR) laser spectroscopic system to classify human cartilage degenerative state.

**Methods:**

Sparse IR spectra of human knee cartilage samples acquired at different contact pressures (i.e., 0.2–0.5 MPa) were analyzed using a variety of chemometric methods (i.e., principal component analysis, balanced random forest, multiple logistic regression). Several data analyses strategies were tested considering different measurement phases (i.e., probe approach vs. sample contact) or cartilage loading i.e., compression vs. relaxation, and relevant spectral characteristics i.e., absorbance or/and slope parameters. In addition, confounding factors were identified ensuring an optimized correlation of sparse IR data with histological evaluation based on osteoarthritis cartilage histopathology assessment system (OARSI grading).

**Results:**

Classification model results using balanced random forest and multiple logistic regression classifier for binary classification of cartilage tissue into healthy (OARSI ≤2) and damaged (OARSI >2) cartilage tissue samples achieved area under the curve (AUC) values of up to 0.86%. Models that combine absorption and slope parameters derived from the fin-shaped measurement signals result in reliable classifications.

**Conclusions:**

Laser-based IR-ATR spectroscopy is a promising analytical technique for future arthroscopic applications facilitating the differentiation between healthy and osteoarthritic cartilage tissue. Acquisition of sparse IR spectra could allow reliable diagnostics if the optical probe is integrated with miniaturized pressure transducers in an arthroscopic probe.

## 1 Introduction

Articular cartilage (AC) has three key functions within the joints: reduction of friction between articulating joint surfaces, protection of subchondral bones from excessive load and redistribution of load during joint movements [1,2]. Degenerative cartilage diseases such as osteoarthritis (OA), which can be caused by progressive cartilage wear with age or post-traumatic degeneration after AC injury may impair these functions and lead to pain, limited mobility, or even complete disability [[3], [4], [5], [6]]. Since degeneration of the AC is an insidious process which may develop over decades [7], it is typically diagnosed at advanced stages once the AC is already significantly degraded or neighboring joint structures (e.g., subchondral bone) are affected by the increasing or otherwise abnormal loads [4,8,9]. Moreover, AC has a slow metabolism with nutrient and water product exchange mainly via the synovial fluid resulting in a rather low self-repairing capacity [5,10]. Consequently, this poses a particular challenge for arthroscopic surgery, as cartilage repair/restoration is performed conservatively to minimize the amount of AC that needs to repair after surgery ensuring that postoperative care/rehabilitation is minimally demanding for the patient. Optical sensor technologies (e.g. infrared or Raman spectroscopy) that can be integrated into endoscopic and arthroscopic diagnostic probes are therefore of interest for the objective localized detection of cartilage degeneration [3,[11], [12], [13]], especially if the probe could be integrated into existing surgical tools or instruments. This would provide the surgeon with live quantitative feedback on early degenerative cartilage changes in areas surrounding visually assessable degenerated tissue and support decision making without significantly modifying existing surgical/diagnostic routines possibly leading to more effective arthroscopic surgery.

Infrared attenuated total reflection (IR-ATR) spectroscopy is a surface-sensitive optical technology that could localize early signs of biochemical degeneration of AC. Chemical and molecular signatures are obtained via evanescent field adsorption upon contact of the probe waveguide with the cartilage enabling IR measurements of water-containing or opaque samples such as AC. Depending on the respective wavelengths of the IR radiation, the penetration depths of the evanescent fields extend several micrometers into the examined sample [14,15]. Since mid-infrared spectroscopy provides access to fundamental molecular vibrations, the identification of chemical components via their ‘vibrational fingerprint’ is straightforward and ideal for *in-situ* monitoring [14,16]. The complex cartilage composition [2,17,18], comprising various chemical components such as water, collagens, and proteoglycans, results in overlapping vibrational signatures, which require multivariate chemometric and machine learning methods to extract qualitative and quantitative information on individual constituents.

The present study follows up and complements previous work by Krebs et al. , which discussed the technical fundamentals of an OA scanner prototype using laser-based IR-ATR spectroscopy. Based on a small set of AC samples, it was demonstrated that the median progression curves vary depending on the cartilage health condition [19]. In this work, the focus is on statistical chemometric analyses of the resulting sparse spectral data, considering the characteristic fin-like laser signal profiles of cartilage, which are potentially linked to the viscoelastic AC properties [2,20,21]. Seven distributed feedback quantum cascade lasers (DFB-QCLs) were applied emitting at key wavelengths relevant for analyzing the condition of AC. Sparse IR data was obtained from experiments performed on human AC plugs measured using different contact pressures (0.2–0.5 MPa) and short measurement windows of approx. 40 s. Firstly, experimentally caused characteristics of AC samples (i.e., cartilage surface structure) were investigated to identify potential confounding factors that need to be considered prior to further data processing using principal component analysis (PCA) on a subset of AC samples. Simultaneously, a variety of strategies for the analyses of the obtained characteristic fin-like laser signal profiles and the experimental conditions were investigated. In a second step, these findings (i.e., confounding factors and different laser signal analyses strategies) were incorporated into predictive models based on balanced random forest (BRF) and multiple logistic regression (MLR) algorithms to achieve the best possible agreement between sparse IR data and histological outcomes assed by the osteoarthritis cartilage histopathology assessment (OARSI) system.

## 2 Methods

### 2.1 AC samples

A dental drill (NTI-Kahla GmbH, Kahla, Germany) equipped with a trephine bit (ID = 4 mm) was used to harvest 244 cartilage sample plugs (central locations of femoral, tibial and patellar tissue) from 17 human cadaver donors obtained from a commercial biobank (Science Care, USA). The cartilage samples were stored in phosphate-buffered saline at −80 °C and thawed only for the experiment and the subsequent histological examination to prevent cartilage degradation. During sample preparation, which mainly consisted of trimming the bone end and during the IR measurements the AC was immersed in Ringer's solution (RS) to protect the tissue from drying and to simulate in-joint conditions.

### 2.2 Laser-based IR-ATR system

**Fig. 1:**
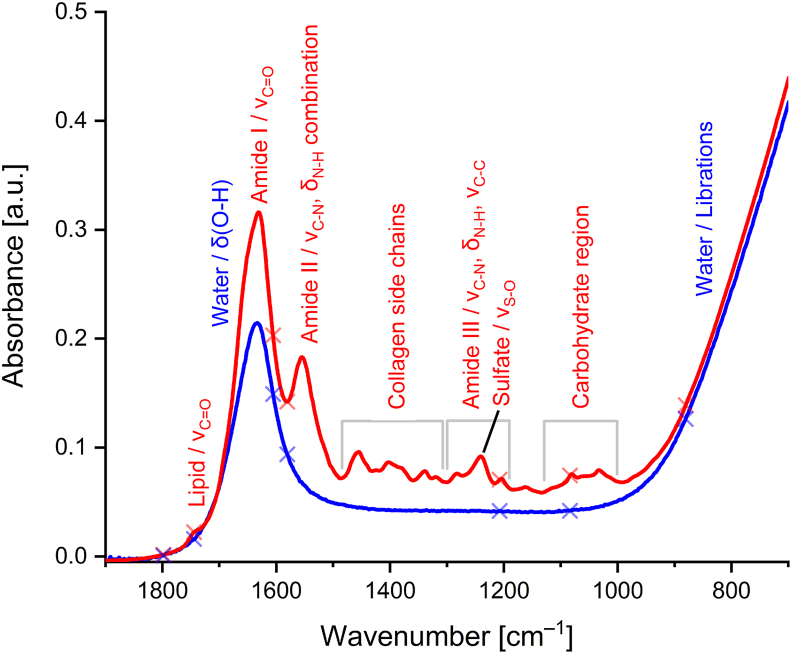
FTIR spectra of articular cartilage (red) and Ringer's solution (blue). The crosses within the spectra mark the wavenumbers that are targeted with the DFB-QCLs of the laser system.

IR data was recorded with a device that combines DFB-QCLs (Nanoplus Nanosystems and Technologies GmbH, Gerbrunn, Germany) with a diamond ATR probe (art photonics GmbH, Berlin, Germany) facilitating future in-joint spectroscopy. IR radiation emitted by seven DFB-QCLs was coupled into a customized polycrystalline silver halide infrared fiber-based beam combiner (art photonics GmbH, Berlin, Germany), guided to the IR-ATR probe and then coupled via a single silver halide fiber to a thermoelectrical mercury cadmium telluride detector (Vigo Photonics, Ożarów Mazowiecki, Poland). The system control electronics (OptoPrecision GmbH, Bremen, Germany) enable simultaneous data acquisition from all lasers at 0.7-s intervals. The DFB-QCLs within the laser system emit at wavelengths 1799 cm^−1^, 1745 cm^−1^, 1606 cm^−1^, 1581 cm^−1^, 1207 cm^−1^, 1084 cm^−1^ and 880 cm^−1^, respectively [19]. The characteristic IR features of AC targeted at these laser emission wavelengths are illustrated and explained in Fig. 1. The diamond ATR probe was connected to the laser unit via F-SMA connectors. The diamond ATR element at the tip of the probe facilitated a sample contact area of approx. 0.45 mm^2^. The cylindrical shaft of the probe had a diameter of 3 mm, a length of 12 cm and was made of medical grade Hastelloy C22. The silver halide fiber cable length between the laser unit and the probe was 70 cm.

The diamond ATR probe was mounted at a fixed position that enabled the cartilage samples to be moved towards the ATR sensor surface via an x-y-z positioner. It was ensured with sample holders (see Fig. S1) that the cartilage and probe surfaces were aligned parallel to each other to achieve optimal contact. An FH-2000 balance (G&G GmbH, Kaarst, Germany) attached on top of the positioner was used to adjust and control the contact pressure between the probe and an osteochondral sample.

Data recording was performed via a custom Lab-VIEW program (OptoPrecision GmbH, Bremen, Germany) that also precisely documents the specific measuring points (e.g., time of target contact pressure, start of sample removal) via manual input. For more details on the device technology and selection of the applied lasers the reader is referred to Krebs et al. [19].

### 2.3 Measurement procedures

Prior to a cartilage measurement, the tip of the diamond ATR probe was immersed in RS for 3 min. Details regarding the surface structure of the cartilage tissue (e.g., smooth/flat, rough, concave, or thick) were visually assessed and documented before the probe was brought into contact with the cartilage to account for potential contact issues with the ATR diamond waveguide. (Further information on this visual assessment can be found in the supplementary information). The cartilage sample was then analyzed during a measurement period of 40 s at a specific contact pressure. After removing the probe, the cartilage sample had 40 s to recover from contact with the probe. The procedure was repeated until triplicate measurements at pressure level of 0.2 MPa, 0.3 MPa, 0.4 MPa and 0.5 MPa, respectively, were obtained. Finally, the RS was analyzed again for 3 min. Strong fluctuations in the RS levels before and after the cartilage measurements indicate changes in the optical system. During the entire measurement procedure, spectral data were recorded at 0.7-s intervals.

### 2.4 Histology and OARSI grading

For the histological examinations, the AC samples were fixed in 4% saline-buffered formaldehyde solution, decalcified with 5% ethylenediaminetetraacetic acid solution. After dehydration, the AC samples were embedded in paraffin. Nine histological sections with a thickness of 3 μm were cut from each sample and stained with Safranin-O, which binds stoichiometrically to the proteoglycans of the AC. Digital images for grading were acquired via a PathScanEnabler-IV (Meyer Instruments, Inc., Houston, USA) using a 40× magnification and a minimum resolution of 100.000× 20.000 pixels with a pixel size of 0.25 μm × 0.25 μm. The degradation assessment was performed according to the OARSI system [22]. Initially, three graders assessed all AC samples individually followed by a group discussion to determine a final assessment score for a sample.

### 2.5 Data analysis

**Fig. 2:**
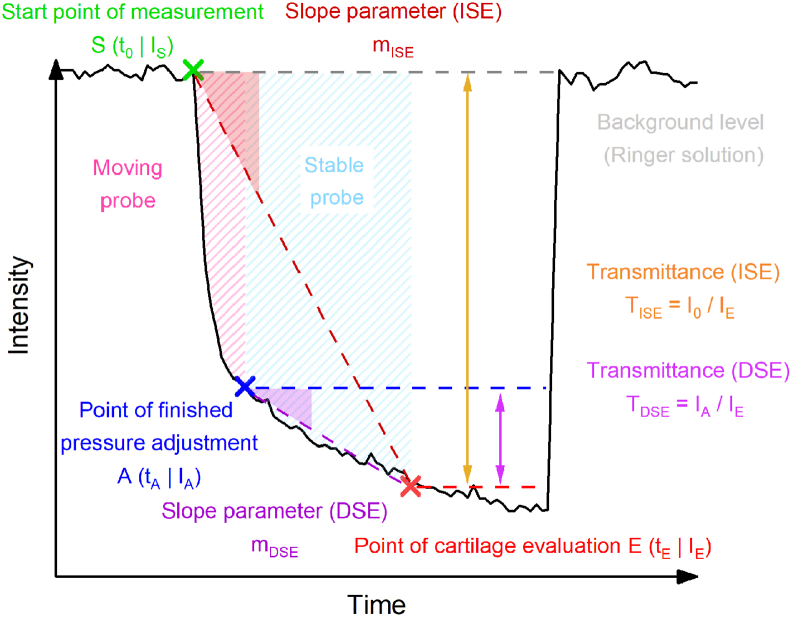
Schematic representation of a cartilage tissue-relevant laser line during a measurement including all specific measuring points and resulting evaluation strategies.

The experimental setup and the viscoelastic contact phenomena between AC and probe lead to the characteristic fin-like laser signal curves shown in Fig. 2 with two main signal domains: (i) an operator-dependent pressure adaptation/cartilage compression phase, whereby a moving system is present until the required contact pressure between IR-ATR probe and cartilage sample is established by the operator, and (ii) an operator-independent phase where the entire system remains stable after applying the targeted contact pressure, i.e., the cartilage tissue adapts/reacts to the applied pressure load. This results in two possible evaluation strategies for the laser signals: (i) evaluation of the change in transmittance between the cartilage and the RS plane and (ii) evaluation of the change in transmittance between the cartilage signal and the signal plane at the time of applied target contact pressure. The first one will be referred to as independent slope evaluation (ISE) while the second as dependent slope evaluation (DSE). The terms ‘independent’ and ‘dependent’ refer to the fact that for DSE an additional sensor system is required to determine the point of pressure adjustment, whereas in the ISE the cartilage condition could be determined via the laser signal changes.

The pattern of the laser signal (‘fin-like pattern’) led to the hypothesis that the laser signal contains not only chemical information, but may also provide information about signal dynamics associated with viscoelastic and contact-dependent behavior of the tissue. Conceptually, contact with the ATR-IR probe can be considered as an impact absorbed by the cartilage tissue, resulting in deformation of the cartilage tissue, which then attempts to slowly return to its original shape. If viscoelastic contact phenomena of tissue were negligible, a step-like laser signal would be obtained. Accordingly, the patterns were analyzed and translated into (i) classical absorbance values and (ii) slope parameters of the laser signal curves. To translate the fin-like laser signal to the absorbance values, the last 5 s of the RS signal as well as the cartilage sample signal were used to calculate the median value of the absorbance. All slope parameters of the laser signals were determined by fitting linear functions to the laser signals. For a measurement duration <5 s, all measured values of the laser signal were used to calculate the median value. For the model building by BRF and MLR classifiers, all outliers and cartilage samples with structural confounding factors (e.g. concave or thick cartilage surfaces) were removed. More details on confounder identification via PCA models can be found in the supplementary information and are shown in Fig. S2. A total of 205 AC samples (84% of total samples) were used, of which 135 samples served as a training set and the rest 70 samples served as a validation set. A binary classification problem was designed to predict whether a cartilage sample is healthy (OARSI ≤2) or degenerated (OARSI >2). The choice of the threshold OARSI = 2 is justified by the lack of completely healthy cartilage samples (OARSI <1) (compare Table S1). The threshold OARSI = 2 allowed binary classification with balanced groups, which is an important condition for the successful classification model building. This cut-off point of OARSI <2 was also used in earlier studies [12,23]. All BRF and MLR models were developed using only the wavelengths of the amide and carbohydrate lasers (1606 cm^−1^, 1581 cm^−1^, 1207 cm^−1^ and 1084 cm^−1^). The MLR model was created using the scikit-learn package in Python. The MLR model was trained with Ridge regularization, also known as L2 regularization, to prevent overfitting, using the default regularization strength parameter (C = 1). The BRF model from the imbalanced-learn (imblearn) package was used with default parameters, except for the number of decision trees, which was increased to 500, and the maximal depth of each decision tree, which was limited to 5. The hyperparameter optimization was not performed, because of the small dataset. The model performance is reported by the area under the curve (AUC) metric, which calculates the overall goodness of the classifier and highlights the potential of the developed method for further investigations. The data were processed such that experimental influences including measurement time, contact pressures between cartilage samples and IR-ATR probe or the different evaluation strategies (i.e., ISE and DSE), which consider the different experimental phases within the cartilage signals may be analyzed and evaluated in more detail. In addition, all analyses were performed using the classical absorbance values, the slope parameters and a combination of both. Details of the OARSI grade distribution of the cartilage samples within the different data sets as well as the classifications according to health conditions are summarized in Table S1. A schematic overview of the workflow used in this study is shown in Fig. S3.

## 3 Results

**Fig. 3:**
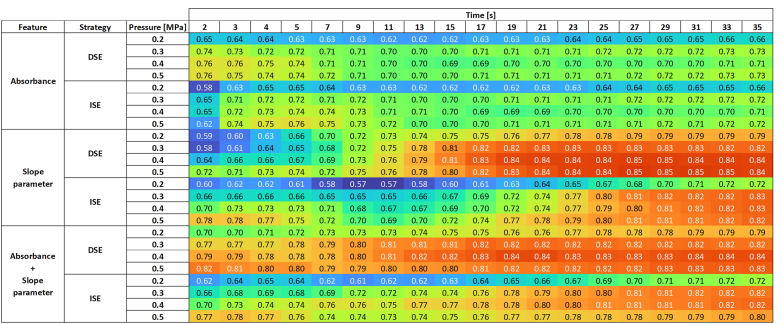
Classification model results using MLR classifier for binary classification of cartilage tissue into healthy (OARSI ≤2) and damaged (OARSI >2) cartilage tissue samples. All results are presented as AUC values.

**Fig. 4:**
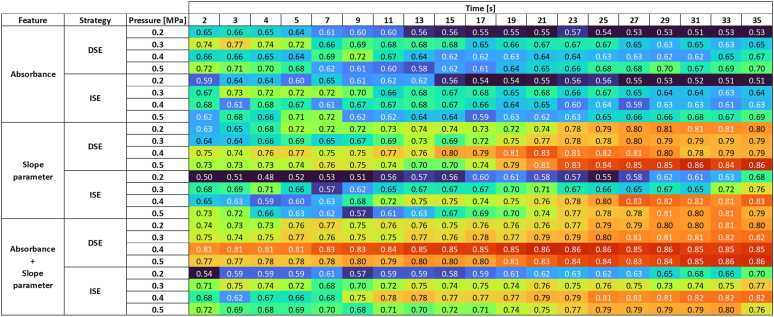
Classification model results using BRF classifier for binary classification of cartilage tissue into healthy (OARSI ≤2) and damaged (OARSI >2) cartilage tissue samples. All results are presented as AUC values.

To study the relationship between IR-ATR measurements and the OARSI grades, BRF and MLR models were used for binary classification of cartilage tissue into healthy (OARSI ≤2) and damaged (OARSI >2) cartilage samples, since those classifiers make different assumptions about the kind of relationship. The MLR method assumes a linearizable relationship [24], while the BRF model assumes a non-analytical relationship based on ‘if-then’ rules [25,26]. The modeling results using both classifiers are shown in Fig. 3, Fig. 4, respectively, represented as heat tables.

The analysis using the absorbance values of the cartilage tissue data shows that contact pressures of 0.2 MPa between probe and sample result in no effective differentiation between healthy and damaged tissue. In MLR and BRF models performed with data obtained at contact pressures of 0.3 MPa or higher, there is a tendency for the prediction accuracy of the models to stagnate or even decrease with increasing measurement time. The decreasing trend is particularly evident in the BRF models. In addition, the different evaluation strategies (ISE and DSE) result in no difference.

The classification using the slope parameters differs greatly from the models using the absorbance values. Even with low contact pressures such as 0.2 MPa, the slope parameters seem to reveal a certain separation behavior between healthy and damaged cartilage tissue samples with increasing measurement time. This can be directly deduced from the MLR and BRF models that were made with the DSE, which can reach AUC values between 0.79 and 0.81 until the end of the measurements. In general, there is a tendency for the ISE to lead to weaker model performance than the DSE. The MLR algorithm also appears to be more effective than the BRF algorithm. The earliest results with AUC values of 0.80 or higher were found within the MLR models, which were built using DSE, with measurement duration of 15 s and contact pressures of 0.3 MPa or higher. Within the MLR models, there is also a trend that higher contact pressures lead to a better classification of degenerative cartilage states. This only vaguely discernible in the BRF models, as their behavior is much more abrupt.

As anticipated, combining absorbance values with slope parameters results in the most effective models, particularly for the short time measurements. Models that were established using the DSE demonstrate AUCs of 0.70–0.82 at the start of measurements. In the BRF and MLR models contact pressures of 0.4 MPa result in the best AUC values. The most effective assessment of cartilage condition can be achieved with BRF models using the DSE, resulting in AUC values up to 0.86 with acquisition times of over 21 s Furthermore, the cartilage degenerative state can be identified more easily with increasing acquisition time, which was also evident in the models established using only slope data. This indicates that the slope data, i.e., information derived from the signal shape, plays a more important role in cartilage assessment than the absorbance data, i.e., information derived from the signal height.

## 4 Discussion

The results obtained by the BRF and MLR prediction models are shown in Fig. 3, Fig. 4. The prediction accuracies regarding the classification of healthy cartilage tissue (OARSI ≤2) and damaged tissue (OARSI >2) are for BRF or MLR models at 0.4 MPa contact pressure evaluated via DSE data considering absorbance values and slope parameters in the range of 78–86%. These results are comparable to the results reported by Virtanen et al. [12] and Tafintseva et al. [27], who both investigated human cartilage using conventional ATR-FTIR spectroscopy achieving prediction accuracies of 66–84%. This also demonstrates that the use of laser-based IR-ATR spectroscopy and the associated sparse spectra combined with chemometric data analysis methods provides comparable results to conventional FTIR spectroscopy, yet, with for intra-operative applications much more practical response times within seconds. The fact that the prediction accuracy depends on the contact pressure and on the data acquisition time is a challenging aspect for future usage during arthroscopic surgery scenarios where cartilage classification should be instantaneous and indicates that further development of the probe is essential. The PCA models in Fig. S4 imply that the clustering dependence on the acquisition time may be caused by significantly damaged cartilage tissue (OARSI >2). Due to the increasing surface damage of cartilage tissue with increasing OARSI grade [22], it is conceivable that a thin layer of water or lubricant is apparently trapped between cartilage tissue and the IR-ATR probe tip. Since healthy cartilage tissue has a higher amide content vs. damaged tissue [12,28], the inclusion of water may lead to a misclassification. The laser emitting at 1606 cm^−1^ is responsible for the identification of healthy tissue and correlates - apart from the amide I and II feature - also with the bending vibration of water (see Fig. 1).

The evaluation via ISE and DSE data and the outcomes of the data analyses (cf. the PCA models in Fig. S2 and the BRF/MLR models in Fig. 3, Fig. 4) indicate the complexity of performing rapid diagnostics of AC via probe-based IR-ATR spectroscopy. Different measurement conditions (i.e., moving vs. stable probe) or not differentiating measurement data from the cartilage compression and relaxation phases (compare Fig. 2) complicates the classification of the cartilage health condition. The relative consideration between different experimental phases (probe contacting vs. steady state) and the consideration of a single pressure domain - as in the case for the DSE data - leads to more efficient classification models, as indicated in Fig. S2d, Fig. 3, Fig. 4. Correlating the late time absorption values against different contact pressures, or normalizing these values, yields results that may indicate dynamic relationships, particularly for the DSE approach (see the supplementary material and Fig. S5 for further details). Therefore, the integration of a miniaturized pressure sensor into the probe appears to be a significant step toward device optimization and usability in real-world scenarios, since this would facilitate the recording of the transition point from the contact phase to the steady state phase to be better tracked during a measurement protocol. Furthermore, a probe with an integrated pressure sensor enables a more precise characterization of the laser profiles regarding the viscoelastic contact phenomena and facilitates the required validation of spectral features related to mechanical cartilage properties through independent mechanical measurements (e.g. confined compression). A direct assessment of the cartilage condition using data acquired when reaching the target contact pressure (see Fig S6) appears to be quite inefficient, as a misinterpretation due to water inclusion between probe tip and cartilage is evident and cannot be compensated via the implemented DFB-QCLs. In turn, this could be compensated by integrating an additional emission wavelength with similar relevance within the chemometric analysis of cartilage – e.g., at 1606 cm^−1^ – that does not coincide with the bending vibration or libration modes of water. The BRF- and MLR-based prediction models (see Fig. 3, Fig. 4) derived from conventional absorbance data also indicate that the selection of relevant laser wavelengths may still be further optimized.

It should be noted though that the problem of misclassified damaged cartilage tissue may be irrelevant during clinical applications, as extensively damaged tissue is frequently recognized by visual inspection (see Fig. S2c/d with S2e/f), i.e., additional confirmation via IR-ATR spectroscopy is not of particular interest. Instead, the discrimination of OARSI grades between 1 and 2 is of particular interest for actual clinical applications. The clustering pattern of samples with OARSI 1.5 and 2 shown in the PCA models (Fig. S2c and S2d) indicates that these states have only minor chemical differences. These grades represent early changes in the cartilage tissue, i.e., the probability is higher that the cartilage defects are not uniform across the probed sample plug. The diamond ATR probe used during this study was able to analyze approximately 3.6% of an AC sample surface during an individual measurement. Additionally, the histological sections considered only approx. 0.1% of the cartilage sample surface area for OARSI grade assessment. Thus, better identification and detection of these OARSI grades could be achieved by increasing the dimension and probing area of the ATR sensor element, more histological sections for the OARSI grading or by using a cartilage assessment strategy that considers significantly more (bio)chemical factors concerning the cartilage tissue conditions beyond the biophysical properties. Furthermore, minute damage caused by the probe due to the ATR element slightly protruding from the probe surface (see Fig. S7) cannot be completely excluded. In subsequent studies, the usage of conical probe tips may lead to less water and lubricant entrapment between the probe and cartilage. In addition, such probes may lead to less steric limitations caused by nonplanar cartilage tissue and a lower contact pressure would be required, which reduces the risk of cartilage damage.

In summary, laser-based IR-ATR spectroscopy and the associated use of sparse IR spectra for the evaluation of cartilage tissue conditions via chemometric classifiers is indeed feasible. The results obtained in this study also indicate that laser-based ATR spectroscopy with high time resolution allows to investigate not only chemical differences in cartilage tissue but may also include physical properties associated with viscoelastic contact phenomena, if the slopes of the laser signals at wavelengths relevant to cartilage are taken into account. This was demonstrated with the slope parameter-based prediction models. Hence, the cartilage condition evaluation via spectral data alone will probably not be sufficient during future clinical usage, the integration of a pressure sensor will certainly increase the reliability and classification accuracy of IR spectroscopic arthroscopes. Additionally, a probe with a pressure sensor may facilitate the fundamental research related to the connection between the flow-shaped laser profiles and cartilage properties. The prediction of OARSI grading based on spectral data is particularly effective in application scenarios once the probe remains steady. As trapped water when contacting the ATR probe with the cartilage may interfere and disturb the modeling results, conical probe tips and the addition of another useful laser wavelength are expected to provide immediate system improvements. Moreover, a more effective cartilage condition assessment strategy should be considered complementing the OARSI approach and being better tailored to IR-ATR spectroscopy to better classify cartilage degenerative states between OARSI grades 1 and 2. For example, the experimental design is being improved in such a way that only the cartilage areas that were in direct contact with the diamond ATR crystal (not with the probe shaft) are histologically examined. The fundamental results presented in this study are groundbreaking for the evolution of laser-based IR-ATR spectroscopic endoscopes relying on sparse IR spectra and appropriate chemometric classifiers toward the in-vivo localization of early cartilage damage during arthroscopic surgery.

## Author contribution

P.K.: Methodology, Conceptualization, Formal analysis, Investigation, Data curation, Writing – original draft, U.B.: Methodology, Conceptualization, Formal analysis, Investigation, Data curation, Writing/review & editing, M.N.: Methodology, Conceptualization, Software, Project administration, Writing/review & editing, V.T.: Methodology, Writing/review & editing, B.Z.: Methodology, Writing/review & editing, V.V.: Methodology, Histological data collection, Writing/review & editing, E.N.: Methodology, Sample preparation, Writing/review & editing, R.S.: Methodology, Sample preparation, Writing/review & editing, I.O.A.: Resources, Conceptualization, Project administration, Funding acquisition, J.T.: Resources, Conceptualization, Project administration, Funding acquisition, Writing/review & editing, S.S.: Conceptualization, Histological data collection, Project administration, Funding acquisition, Writing – review & editing, A.K.: Methodology, Resources, Project administration, Funding acquisition, B.M.: Conceptualization, Resources, Writing – review & editing, Supervision, Project administration, Funding acquisition.

## Ethics

The data used in this work is based on experimental studies conducted as part of the European Union's Horizon 2020 research and innovation program ‘MIRACLE’ (project grant agreement No 780598). The activities there were approved by the Research Ethics Committee of the Northern Savo Hospital District (Kuopio University Hospital, Kuopio, Finland, permission #134/2015).

## Role of the funding source

This research was funded by the 10.13039/501100000780European Union's 10.13039/501100007601Horizon 2020 research and innovation program (H2020-ICT-2017-1, MIRACLE project grant agreement No 780598). The study was also supported by the Ministry of Economics, Labour and Tourism of the Federal State of Baden-Württemberg, Germany (BW1_4109/03) and the 10.13039/501100002341Academy of Finland (project number 315820).

## Conflicts of interest

The authors declare no competing financial interest.
