# Supplementary material for: Assessment of degenerative states of articular cartilage via sparse laser-based mid-infrared attenuated total reflectance spectroscopy
Source: Osteoarthr Cartil Open. 2026 May 30;8(3):100826. doi: 10.1016/j.ocarto.2026.100826 (PMC13277485; doi:10.1016/j.ocarto.2026.100826)
Supplement: Multimedia component 1 [file mmc1.docx]

Supplementary Information

**Assessment of Degenerative States of Articular Cartilage via Sparse Laser-Based Mid-Infrared Attenuated Total Reflectance Spectroscopy**

P. Krebs ^1^, U. Blazhko ^2^, M. Nägele ^3^, V. Tafintseva ^2^, B. Zimmermann ^2^, V. Virtanen ^4^, E. Nippolainen ^5^, R. Shaikh ^5^, I.O. Afara ^5^, J. Töyräs ^5,6,7^, S. Saarakkala ^4,8^, A. Kohler ^2^ and B. Mizaikoff ^1,9*^

^1^ Institute of Analytical and Bioanalytical Chemistry, Ulm University, Ulm, Germany

^2^ Faculty of Science and Technology, Norwegian University of Life Sciences, Ås, Norway

^3^ OptoPrecision GmbH, Bremen, Germany

^4^ Research Unit of Health Sciences and Technology, Faculty of Medicine, University of Oulu, Oulu, Finland

^5^ Department of Technical Physics, University of Eastern Finland, Kuopio, Finland

^6^ Science Service Center, Kuopio University Hospital, Kuopio, Finland

^7^ School of Electrical Engineering and Computer Science, The University of Queensland, Brisbane, Australia

^8^ Department of Diagnostic Radiology, Oulu University Hospital, Oulu Finland

^9^ Hahn-Schickard, Ulm, Germany

**Visual inspection of articular cartilage samples**

The preliminary visual inspection of the cartilage samples was performed for purely technical reasons, to ensure optimal contact with the ATR diamond waveguide. Since the probe shaft (3 mm) is significantly wider than the diamond (0.8 mm), uneven surfaces could prevent the ATR sensor from making proper contact with the cartilage. They are classified as follows:

1. Smooth/flat: A flat cartilage surface that is expected to allow optimal contact between the ATR crystal and the cartilage tissue.
2. Rough: Minimal irregularities are visible but contact between the ATR crystal and the cartilage tissue is possible without any problems.
3. Thick: Cartilage layer thicker than the cartilage plug diameter (4 mm); contact appears possible without issues, but the cartilage tissue showed a tendency to tilt to the side.
4. Concave: These samples were rated critically. There was a risk that the wide probe shaft would lie on the tissue without the diamond ATR crystal being in contact with the cartilage, resulting in measurements taken with insufficient pressure and making deviations compared to the other classes. Such samples can be easily identified just before contact, when the probe and the cartilage surface are in close proximity.

**Articular cartilage sample holders**

Sample holders with 4 mm V4A stainless steel tubes were created for performing the IR measurements of cartilage samples. For cartilage samples with crooked bone pieces samples holders with M4 V4A stainless steel washers were created. The tubes or M4 washers were glued to the Petri dishes using a cyanoacrylate-based Loctite 401 instant glue (Henkel AG & Co. KGaA, Düsseldorf, Germany). The cartilage samples were placed within the 4 mm tubes, while the Petri dishes were filled with Ringer's solution. Thus, the samples were permanently protected from drying out and the cartilage surfaces could be easily aligned parallel to the ATR element, ensuring ideal contact. The fact that the samples were not glued directly to the Petri dishes also avoided the risk of adhesive residue getting on to the cartilage surface and influencing the IR measurements.


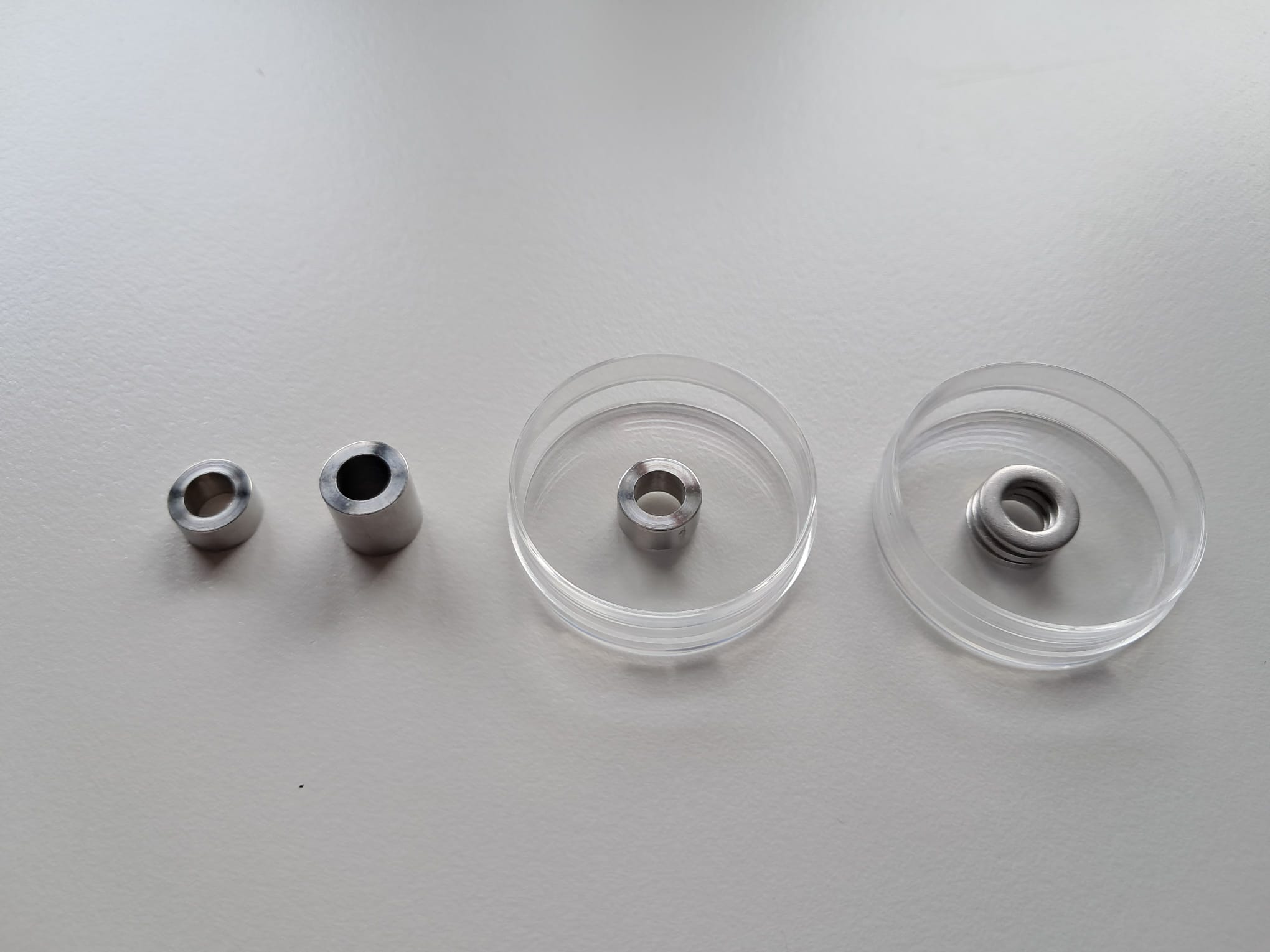


**Fig. S1** Sample holders for cartilage samples with a V4A stainless steel tube with an inter diameter of 4 mm (left) and M4 V4A washers (right).

**Principal component analysis for confounder identification**

A reduced cartilage sample data set (n = 35: harvested from 12 cadavers) consisting of absorbance values was examined using principal component analysis (PCA). Only cartilage samples that had a consistent grading result regarding the individual graders and the subsequent grader discussion were used, and thus, should be ideal representatives of their respective OARSI grade. The spectral data were analyzed separately according to the pressure levels (0.2 MPa, 0.3 MPa, 0.4 MPa and 0.5 MPa) and acquisition times (10 s, 20 s, 30 s and 40 s) using ISE and DSE. Sparse spectra that showed erroneous measurements (e.g. insufficient contact with the AC sample) were removed by visual inspection from the data set. In the case of the reduced data set, 3 out of 435 measurements were excluded. The data processing was kept minimal and comprised only mean centering of the data. The data acquired at a wavenumbers 1581 cm^–1^ and 1745 cm^–1^ were excluded.^.^ Data of 1581 cm^–1^ showed no significant influences within the PCA models, while 1745 cm^–1^ was excluded due to avoid falsification by small fat droplets within the RS originating from the bone material. The obtained PCA models were built to provide initial insight into possible confounding factors and the general pattern in the data. The PCA models were developed using a combination of MatLab R2019a (The MathWorks, Inc., Natick, USA) and Eigenvector Toolbox 8.7 (Eigenvector Research, Inc., Manson, USA).

The PCA models obtained for both ISE and DSE, for data acquired using measurement periods of 20 seconds at a contact pressure of 0.4 MPa are shown in Figs. S2a and S2b. In this PCA analysis, two principal components (PC) were selected that describe 95.68% of the variation within the data. Signs of clustering were observed for contact pressures of 0.4 MPa and higher, as shown in Fig. S8. It is evident that the damaged cartilage condition overlaps with the healthy cartilage condition and occurs within the first few seconds - especially at lower contact pressures - which appears to decrease with time, as can be seen in Fig. S4. It is immediately apparent that the separation of the different cartilage health states achieved within PCA models is not ideal, although only data from cartilage samples that were clearly assigned to an OARSI grade were used. With regard to clustering, the healthy and intermediate classes appear to be better distinguishable within 10 to 20 seconds from the other classes, while the intermediate classes can be distinguished from the damaged classes in 40 seconds.

In the PCA performed using ISE (see Fig. S2a), the cartilage health classes seem to separate in a V-shaped pattern, whereby the healthy state separates from the intermediate state along PC 1. The damaged state emerges along PC 1 and PC 2. Concerning the loadings (see Fig. S2g), the changes along PC 1 are related to spectral changes of the amine signal at 1606 cm^–1^, while the separation along PC 2 can be correlated to signal changes of the laser emitting at 1084 cm^–1^, i.e., carbohydrates. Considering the data points in terms of their OARSI grades (Fig. S2c), no sequential separation behavior according to increasing OARSI grades is evident. Instead, the PCA score plot seems to represent structural effects of the cartilage tissue (see Fig. S2e), as especially the intermediate cartilage state broadens due to varying cartilage structures. Concave cartilage surfaces that are more difficult to contact with a cylindrical ATR probe appear in the fourth quadrant of the scores plot. In contrast, samples with thick cartilage layers tend to cluster in the second quadrant of the scores plot. Samples at an intermediate health state with a smooth or roughened cartilage surface are located in between the concave and thick samples.

The PCA analysis performed using DSE data (see Fig. S2b) results in a more distinct clustering of the different cartilage conditions, especially between intermediate and damaged tissues. Considering the OARSI grade (Fig. S2d), a sequential separation from grade 1 to grade 1.5 or 2 from the fourth to the first quadrant of the score plot is noticeable. Grade 2.5 is in the second quadrant and cartilage samples with grade 4 are in the third quadrant. Only samples with grade 4.5 appear to show no distinct trend along PC 1 but rather along PC 2. The loadings of the PCA calibration (see Fig. S2h) indicate similar behavior to the previous model for the ISE data, whereby healthy cartilage tissue shows high amide signals (1606 cm^–1^) and lower carbohydrate signals (1207 cm^–1^). The opposite trend is observed for damaged cartilage samples. This model also reveals significant scattering of concave cartilage samples, while thick cartilage samples show as a separate group in the score plot (compare Fig. S2f).


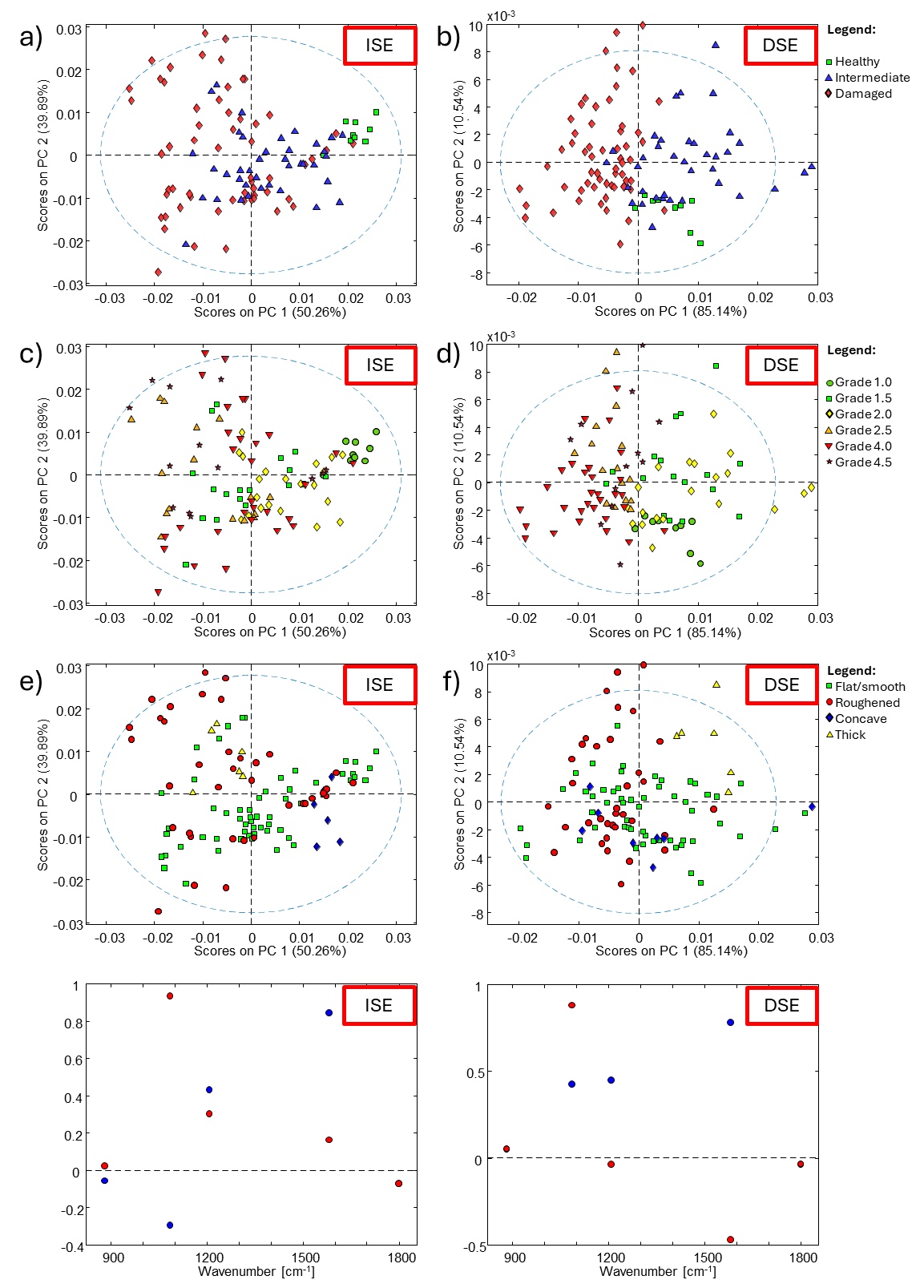


**Fig. S2** PCA models obtained with the reduced cartilage data set using absorbance values obtained via ISE (left) and DSE (right). The data was captured after 20 seconds measurement time and a contact pressure of 0.4 MPa. a)/b) Visualization of the separation potential according to different states of health c)/d) Visualization of the separation potential according to different OARSI grades e)/f) Influences of the different cartilage structures. g)/h) Respective loadings of PC1 (blue points) and PC2 (red points).

**Table S1** OARSI grading distribution and health state classification within the cartilage data sets.

|  | **Reduced cartilage dataset**  **(n = 35)** | | **Cartilage training dataset (n = 135)** | | **Cartilage validation dataset (n = 70)** | |
| --- | --- | --- | --- | --- | --- | --- |
| **Grade** | **Samples** | **Class** | **Samples** | **Class** | **Samples** | **Class** |
| 0 | 0 | Healthy | 0 | Healthy | 0 | Healthy |
| 1 | 3 | Healthy | 5 | Healthy | 2 | Healthy |
| 1.5 | 6 | Intermediate | 22 | Healthy | 5 | Healthy |
| 2 | 7 | Intermediate | 20 | Healthy | 13 | Healthy |
| 2.5 | 5 | Damaged | 32 | Damaged | 19 | Damaged |
| 3 | 0 | Damaged | 1 | Damaged | 1 | Damaged |
| 3.5 | 0 | Damaged | 3 | Damaged | 0 | Damaged |
| 4 | 10 | Damaged | 28 | Damaged | 13 | Damaged |
| 4.5 | 4 | Damaged | 19 | Damaged | 17 | Damaged |
| 5.0 | 0 | Damaged | 2 | Damaged | 0 | Damaged |
| 6.5 | 0 | Damaged | 3 | Damaged | 0 | Damaged |

**
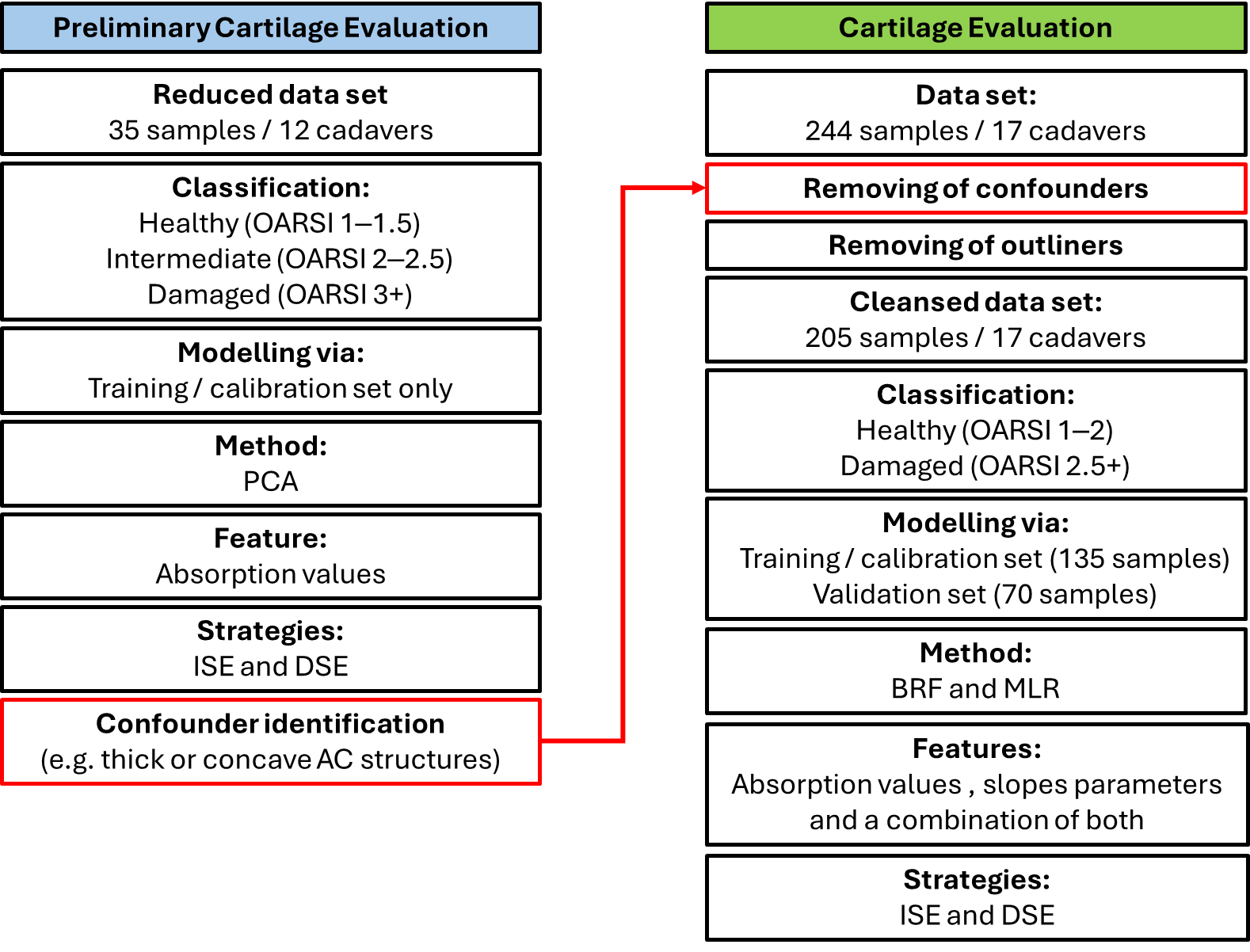
**

**Fig. S3** Schematic diagram of the workflow used in this work for data evaluation of the cartilage samples.

**Fig. S4** PCA score plots for the models built using ISE data measured at 0.4 MPa contact pressure after different time intervals. The classification is according to healthy (OARSI 0–1, green squares), intermediate (OARSI 1.5–2; blue triangles) and damaged cartilage tissue (OARSI 2–4.5, red diamonds).**
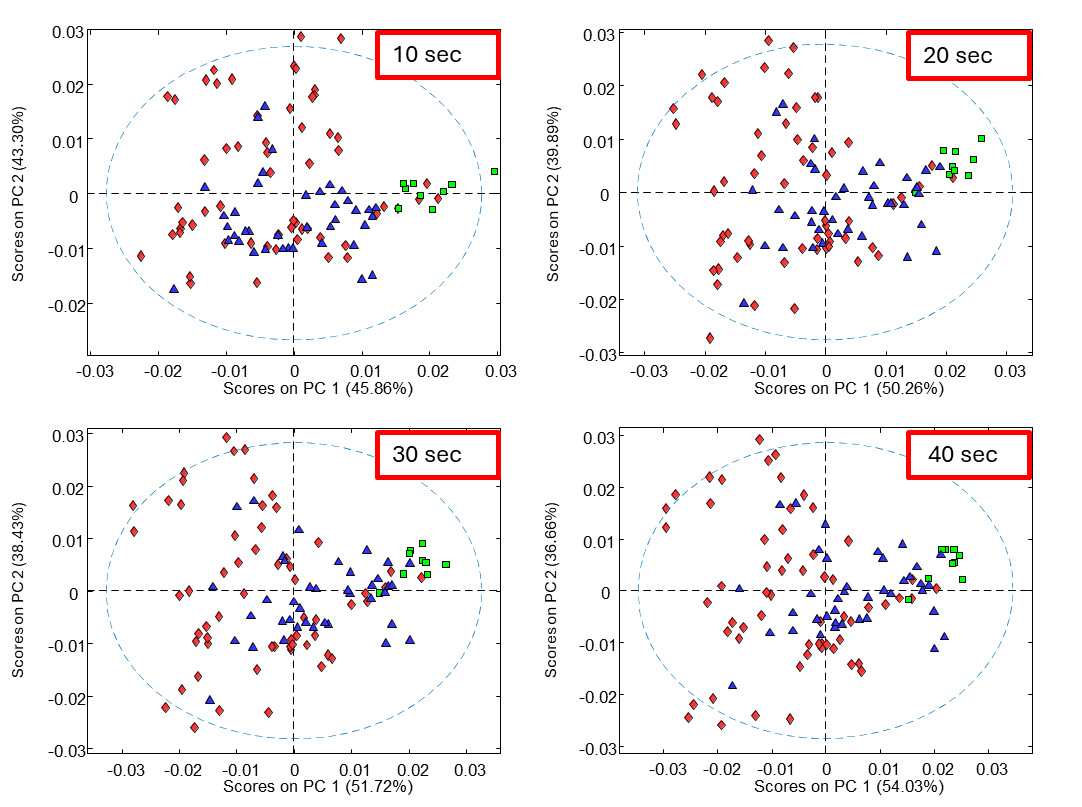
**

**Indication of dynamic contact phenomena**

To investigate the relationship between absorbance and different contact pressures, the absorption data was taken from the reduced dataset, which was also used for the PCA models (Fig. S2). In order to have data disentangled from transient viscoelastic effects, absorption data collected after a measurement period of 30 seconds were used. Figure S3 shows the absorption behavior depending on the contact pressure for the wavenumbers 1084, 1207 and 1606 cm⁻¹. For this purpose, the ISE and DSE approaches were both used, including the respective normalizations (where the value was divided by the corresponding contact pressure). The individual approaches and results are explained in more detail below.

***Signal response: contact pressure vs. absorbance (using the ISE approach)***

The amide bands (1207 cm⁻¹ and 1606 cm⁻¹) show clear patterns in relation to healthy, intermediate and damaged tissue. The signals resulting from healthy/intermediate tissue are more intense and indicate a higher amide content, i.e., the tissue structure is apparently intact. Damaged tissue has lost its cartilage integrity, which is reflected in the lower amide content. The higher carbohydrate content could be related to a higher demand for lubrication, as damaged tissue has a larger surface area vs. healthy tissue with carbohydrates emerging from within the tissue being released to the outside. As the respective intensities no longer increase beyond 0.5 MPa, the pressure limit was set at this point to avoid cartilage damage.

***Normalized signal response: contact pressure vs. absorbance (using the ISE approach)***

By normalizing the signal (i.e., dividing the measured intensities by the contact pressure), a similar behavior for all relevant wavelengths is obtained. Interestingly, as the pressure increases, the signal intensity decreases, which appears counterintuitive. The striking aspect here is that across all wavelengths a similar signal drop from 0.2 (at 0.2 MPa) to <0.1 (at 0.5 MPa) is observed. The only chemical explanation across all wavelengths is behavior of water. Since water is a stronger IR absorber vs. tissue, the loss of intensity is hypothesized to result from a redistribution of water within the cartilage within the evanescent field with increasing pressure.

***Signal response: contact pressure vs. absorbance (using the DSE approach)***

The change in intensity at 1084 cm⁻¹ following the completion of the probe calibration does not show any significant pressure dependence. The elevated values observed in intermediate and damaged tissue could indicate the presence of lubricants or carbohydrates, as previously discussed. However, damaged tissue should indeed show higher values vs. intermediate samples. The amide bands at 1207 cm⁻¹ and 1606 cm⁻¹ exhibit a behavior similar to that observed with the ISE. The intensity changes after probe adjustment indicate whether a fin-shaped signal has formed or whether the curve has developed rather flatly, which could possibly be traced back to cartilage integrity.

***Normalized signal response: contact pressure vs. absorbance (using the DSE approach)***

If the values are standardized against the respective contact pressures from the DSE approach, the behavior for the amide bands appears more dynamic. Damaged tissue exhibits a rather flat curve suggesting that the tissue does not significantly respond to the contact pressure, while healthy tissue shows a steep curve that decreases as the contact pressure increases. If this behavior correlates with the redistribution of water within the tissue (i.e., decrease of the IR water signature), viscoelastic effects could indeed be tracked via IR-ATR spectroscopy.





**Fig. S5** Absorption values of wave numbers 1606, 1207 and 1084 cm⁻¹ after 30-second measurements of cartilage samples. The results of the ISE and DSE analyses are shown based on raw data and normalized data (by dividing through contact pressure). The classification is according to healthy (OARSI 0–1, green bars), intermediate (OARSI 1.5–2; blue bars) and damaged cartilage tissue (OARSI 2–4.5, red bars).

**
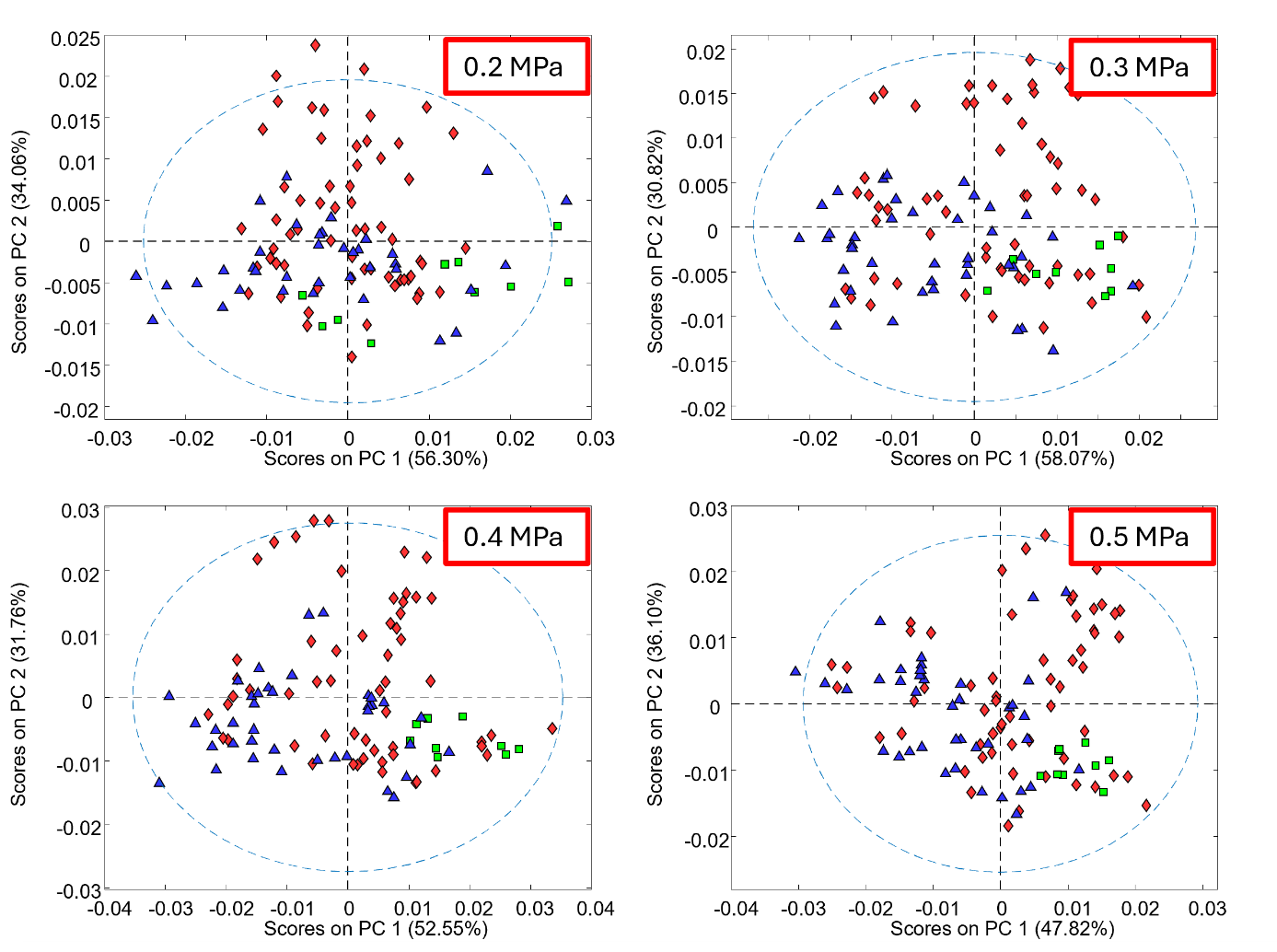
**

**Fig. S6** PCA models of data measured exactly at the target pressure. The classification is according to healthy (OARSI 0–1, green squares), intermediate (OARSI 1.5–2; blue triangles) and damaged cartilage tissue (OARSI 2–4.5, red diamonds).


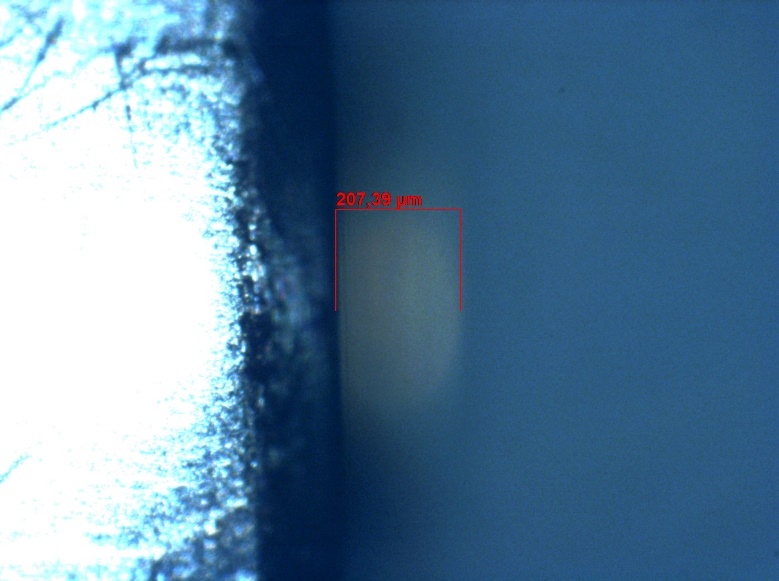
**Fig. S7** Microscopic image of the ATR probe tip indicating a diamond waveguide protruding out of the plane.


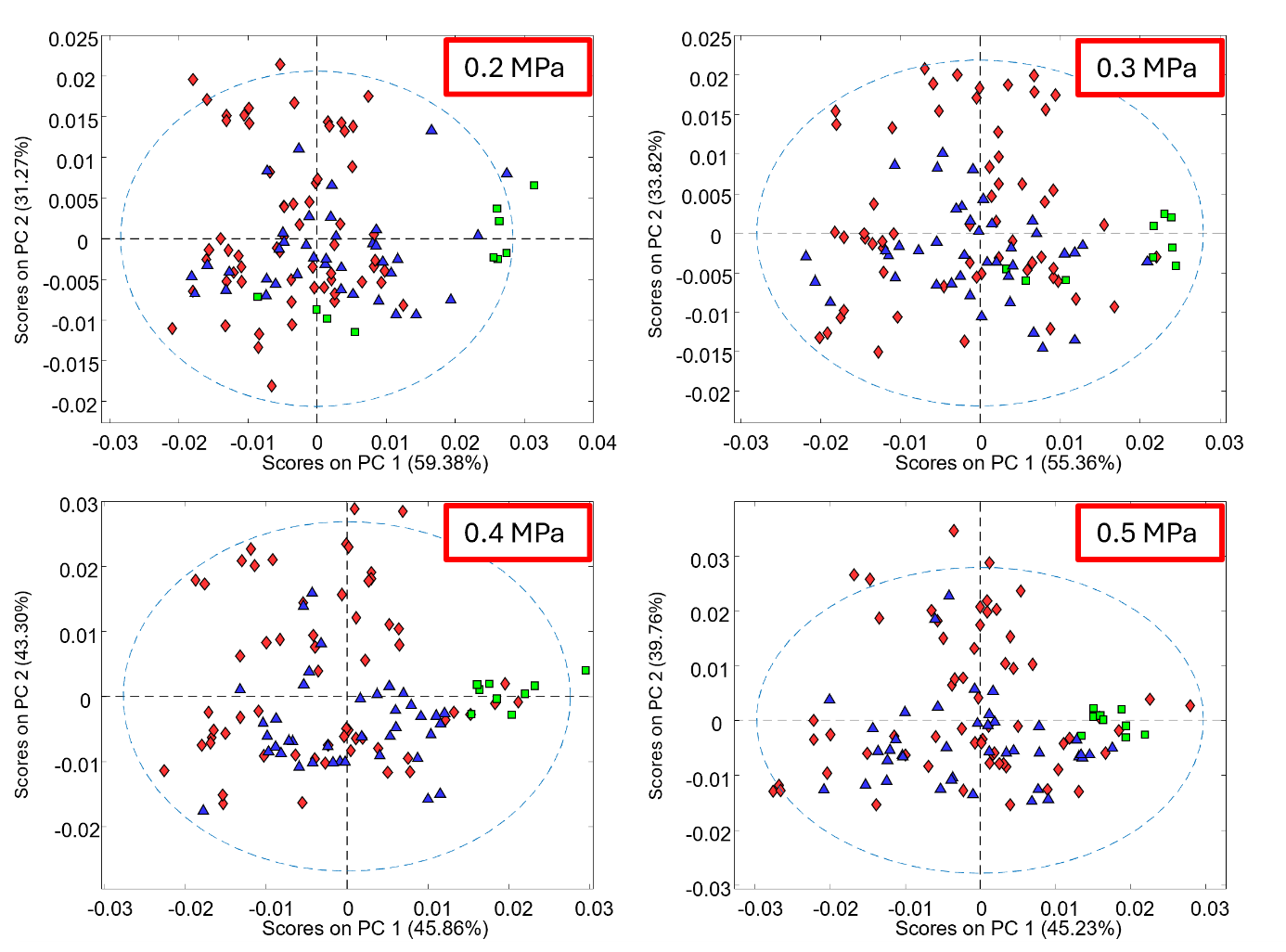


**Fig. S8** PCA score plots for the models built using ISE data measured after 10 seconds under different contact pressures. The classification is according to healthy (OARSI 0–1, green squares), intermediate (OARSI 1.5–2; blue triangles) and damaged cartilage tissue (OARSI 2–4.5, red diamonds).
